# Study protocol of a mixed method pragmatic quasi-experimental trial to evaluate the day activity services targeted at older home care clients in Finland

**DOI:** 10.1186/s12877-022-03512-7

**Published:** 2022-10-21

**Authors:** Hanna Ristolainen, Leena Forma, Jemma Hawkins, Elisa Tiilikainen

**Affiliations:** 1 University of Eastern Finland, Yliopistonranta 1, 70211 Kuopio, Finland; 2 Laurea University of Applied Sciences, Ratatie 22, 01300 Vantaa, Finland; 3 Faculty of Social Sciences and Gerontology Research Center (GEREC), Tampere University, Kalevantie 4, 33100 Tampere, Finland; 4 Cardiff University, Spark, Maindy Road, Cardiff, CF24 4HQ UK

**Keywords:** Day activity service, Home care, Social inclusion, Quality of life, Loneliness

## Abstract

**Background:**

In Finland, the ‘day activity service’ is targeted at older home care clients who are unable to participate in other available activities due to poor health or functional disabilities. The aim of the day activity service is to support home care client’s ability to live at home and to enhance their wellbeing and social inclusion. This mixed method study examines the effectiveness, cost-effectiveness and process of the day activity service.

**Methods:**

The target sample size is 200 participants. The intervention group (*n* = 100) is composed of home care clients who begin to participate in the day activity service. The comparison group (*n* = 100) are home care clients who do not participate in the day activity service, and whose functioning and care needs are similar to the participants of the intervention group. The primary outcome is social inclusion (ESIS-scale). Secondary outcomes are loneliness (single item and De Jong Gierveld Loneliness Scale) and social care related quality of life (ASCOT). Baseline, three-month and six-month follow-up surveys are gathered from intervention and comparison group participants in order to compare outcomes between groups pre- and post-intervention.

Costs of health and social services, based on administrative data, and the costs of the intervention are utilized in examining the cost-effectiveness of the intervention with the above-described measurements. Qualitative data are collected by interviewing the intervention participants (*n* = 10) and professionals working at the day activity centres and older people’s services (4 focus groups) to explore the perceived outcomes and process of the intervention to find out how and why the intervention is effective or ineffective.

**Discussion:**

The study seeks to produce a comprehensive understanding of the effectiveness, cost-effectiveness and implementation process of the day activity service.

**Trial registration:**

ISRCTN13146087, Registration date 03/04/2022.

## Background

In recent decades, living independently has become high valued and guiding principle of ageing policies [1]. Consequently, in Finland and in other European countries, home-based care has become the primary service of supporting older people’s daily life. At the same time, low resources in formal home care and older home care client’s increased disadvantages and care needs have led to experiences of the inadequacy of support and services [2, 3]. Even though living in familiar surroundings has many positive consequences [4], residential stability may not always be beneficial if the older person is bound to a place where wellbeing and sense of security are challenged in different ways [5, 6].

In the Finnish legislation, municipalities or federations of municipalities (after January 2023: wellbeing service counties) have been obligated to organize home care for older people [7, 8], but the producers of these care services may also be private companies and third sector organizations. Formal home care includes personal or virtual visits and support services, such as meal services, cleaning services and safety services. Formal home care has focused mostly on clients’ physical needs and resources [9], while home care clients have unmet needs related to social activities [10], which, combined with limited and inadequate home care services, may lead to different forms of social exclusion. In Finland, social exclusion of older home care clients has been addressed by a group-based intervention called *day activity service*. The day activity service is an additional non-statutory service produced mainly by municipalities or federations of municipalities.

The day activity service has been widely used in formal home care services, yet systematic research evaluating the service in the Finnish context has been non-existent. The day activity service is often conducted in day centres where other activities are also available for older people who are not in need of formal home care. Previous studies in other countries have shown that day centres may be beneficial in supporting older people to live at home for longer [11]. However, little is known about the effectiveness and cost-effectiveness of day activity groups targeted at older home care clients.

The primary aim of the day activity service is to support home care clients living at home by promoting their health and wellbeing, maintaining their physical, psychological, and social functional ability, and enhancing social inclusion. As this study is part of the SOLDEX (Old-age social exclusion in home care: Prevalence, meanings and intervention) project [12], we focus on examining the day activity service from the perspective of social inclusion. In addition, we are interested in the social functional ability and comprehensive wellbeing related to the day activity service.

The study is carried out following the UK Medical Research Council framework of the evaluation of complex interventions [13, 14]. The day activity service can be defined as a complex intervention; therefore, we systematically evaluate both effectiveness and process of the intervention (see [14]). The study focuses on examining the outcomes of the intervention in terms of social inclusion, loneliness and social care related quality of life. The effectiveness and cost-effectiveness will be examined using a pragmatic quasi-experimental design because the day activity service is already in use. By integrating qualitative methods into the trial design, we plan to investigate how and why the intervention works or does not work more in depth (see [15]). From the perspective of process evaluation, it is possible to examine several issues such as the implementation of the intervention or mechanisms of impact [16]. In this study, we will focus on the process of the intervention delivery by examining facilitators and barriers of its implementation. The program theory of the day activity service is somewhat unknown; thus, the process evaluation will also explore understandings of the program theory [14, 17]. This will help to develop an understanding of how the intervention might be implemented in other contexts, if shown to be effective. In addition, we will examine perceived and unexpected outcomes both quantitatively and qualitatively.

### Objectives

The general aim of the study is to produce knowledge on the effectiveness, cost-effectiveness and the implementation process of Finnish day activity services targeted at older home care clients. The specific research questions are:

1. What are the effects of the day activity service in terms of social inclusion, loneliness, and social care related quality of life?
2. What are the costs and cost-effectiveness of the day activity service compared to care without the day activity service?
3. What are the facilitators and barriers related to the implementation of the intervention?
4. What are the perceived and unexpected effects of the day activity service?

## Methods

### Overall study design and setting

**Fig. 1:**
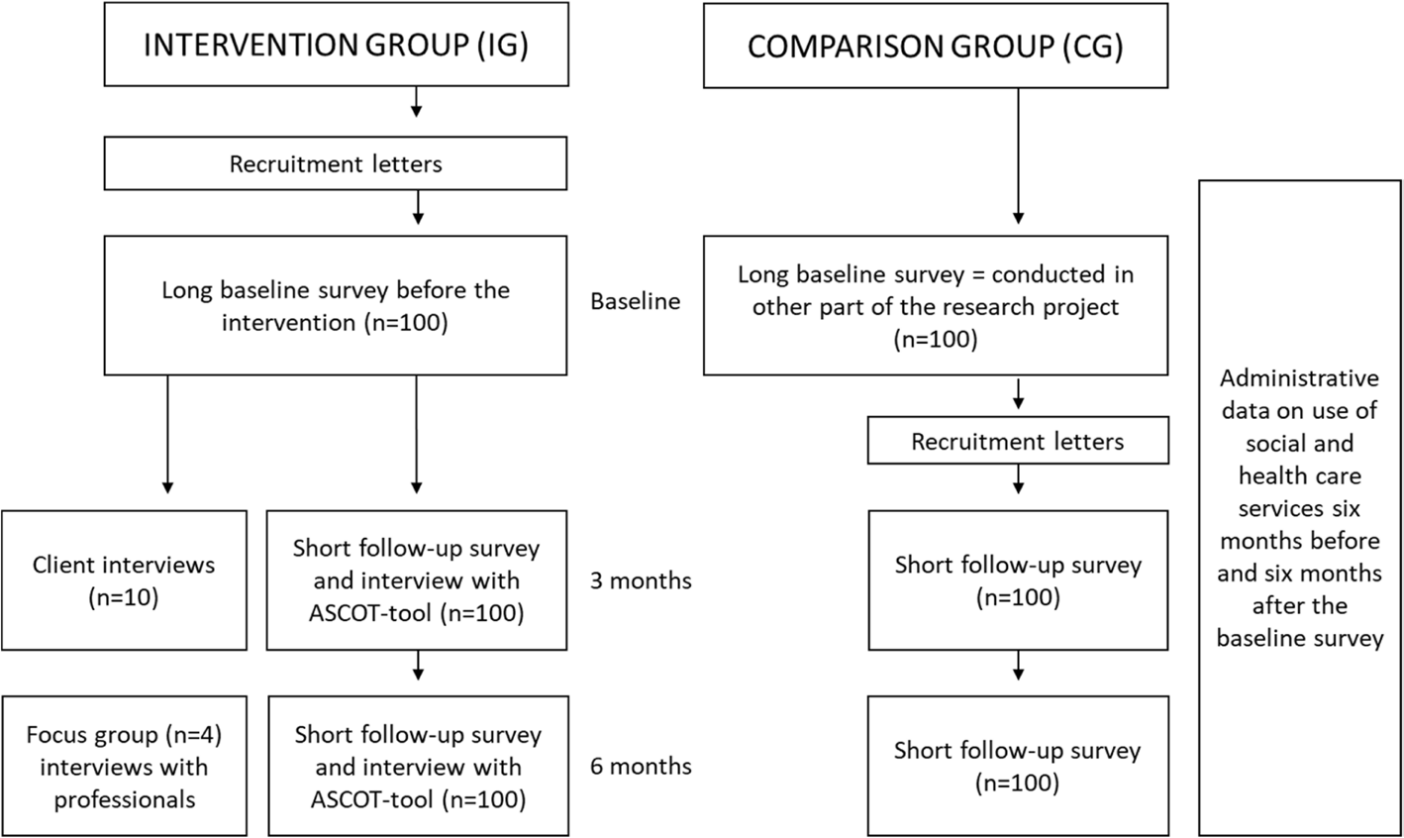
Flow diagram of the study design

This mixed method pragmatic quasi-experimental trial will examine the effectiveness, cost-effectiveness, and process of an existing intervention called *day activity service*. The overall study design is described in Fig. 1. The effectiveness is evaluated with quantitative quasi-experimental design by comparing a group of home care clients taking part in the intervention (IG) with a group of home care clients with similar characteristics not taking part in the intervention (CG). The study is a pragmatic trial, as the day activity service is an existing intervention carried out in ordinary circumstances as part of formal home care services [18]. The trial is quasi-experimental, because randomization is not an option due to the fact that the intervention is already in use. Baseline, three-month follow-up, and six-month follow-up surveys are collected from IG and CG participants. The cost-effectiveness evaluation is based on the quasi-experimental trial design and the administrative data of the social and health care service use. The process evaluation is mainly based on qualitative data that is collected by interviewing the IG participants and the professionals working at the day activity service and formal home care.

The study is conducted in Finland in one municipality and one social welfare and health care joint authority consisting of seven municipalities. There are approximately 2300 older home care clients in the research areas. As of January 2022, 210 of the older home care clients in the research areas had been granted access to the day activity service. According to the professionals responsible for the day activity service, the number of participants has somewhat reduced during COVID-19 pandemic. The research areas include both urban and rural residential districts. The day activity service is carried out across 12 municipalities, villages, or residential areas.

### Description of the intervention

The intervention is targeted at older home care clients who are not able to participate in other activities because of their poor health or functional ability. The day activity service is a complex intervention which means that it has multiple aims and there is variation in how it is carried out in different settings. The principle aim of the day activity service is to support the older home care clients’ ability to live in their own homes by promoting their health and wellbeing, maintaining their physical, psychological, and social functional ability and by enhancing social inclusion. More specifically, the intervention has aims to; maintain memory, promote social interaction, provide peer support, and reduce loneliness and sense of insecurity.

The day activity service is an additional service to the regular home care services and takes place in local day activity centres or other places once a week. It is a group-based intervention, and the duration of one meeting is from 2 to 6 hours. The intervention includes rehabilitative and preventive activities such as physical exercising, outdoor activities, thematic discussions, activating memory, listening to music, having lunch and coffee together with group participants. The day activity service is tutored by practical nurses or home helpers. In the study sites, there are day activity groups of 2 hours, 3 hours, 4 hours, and 6 hours. There is also variation in the content and activities of the intervention. A payment per meeting is between 5 to 23 euros depending on the duration of the service. Transport is arranged if necessary, paying for a local bus ticket.

Home care clients need to apply for the service and get a service decision on it to take part in the day activity service weekly. The criteria for the decision are i) not able to participate in other activities produced by municipalities, private sector or third sector, and ii) is capable of attending group activities. Severe memory disorder can prevent participation, for example.

### Quasi-experimental trial

#### Participants, selection criteria and recruitment

The target sample size for the intervention study is 200 participants (100 IG + 100 CG) based on previous research with similar study designs (e.g., [19]) and directional sample size calculations. We accepted an alpha risk of 0.05% and a power of 80% at an effect size of 0.5. A theoretical sample of 64 participants in the IG and 64 participants in the CG will be needed. Finally, we assumed that potential attrition rate may be 20–30% due to the functional decline of the participants.

The IG is composed of home care clients aged 65 years or older who are new participants in the day activity service. All new participants of the day activity service are eligible for the IG. The participants are recruited with the help of personnel who are responsible for the official decisions of the day activity service. Personnel or researchers will call older people in receipt of service decisions to access the day activity service and invite them to participate in the study. Researchers will receive the contact details of the day activity service participants from the personnel. Information about the study, an informed consent form and the first questionnaire are sent to those home care clients who agree to participate in the study when calling them. IG participants fill the informed consent form and baseline survey before beginning to participate in the day activity service.

The CG consists of home care clients aged 65 years or older who do not participate in the day activity service, and whose functioning and health are similar to the participants of the IG. Possible reasons for non-participation include being on the waiting list and not knowing about the service. The CG participants are recruited from the sample of home care clients who attend the other part of the research project from May to October 2022 (see [12]). The sample (target *n* = 1700) of home care clients respond to the same survey used as baseline survey in the quasi-experimental trial. The formation of the CG proceeds in three steps. 1) Potential CG participants are chosen based on the questions and measurements of the survey. The primary indicator is the question: Do you participate in group activities or hobby activities? Those who respond “No, but I would like to participate” are selected as potential CG participants at first stage. 2) After that, the potential respondents for the CG are compared with the respondents who have recently started to attend the day activity service. Comparison is conducted using general indicators related to their functioning and health, such as quality of life (Euro-HIS-8) and perceived health. 3) The invitation and follow-up surveys are sent to 130 home care clients whose functioning and health are as similar as possible to those clients attending the day activity service.

The recruitment of IG participants begins in August 2022 and continues until the target sample size is achieved. The estimated recruitment rate is 14 new study participants per month; therefore the recruitment phase may last until March 2023.

#### Study conduct

At the beginning, the study participants will complete a long questionnaire (baseline survey). The CG participants will fill their baseline survey as part of the overall study (SOLDEX project). After responding to the baseline survey, IG participants will begin to attend the day activity service meetings once a week. The CG participants will receive home care services as usual and other social and health care services if needed. The three-month and six-month follow-up surveys will be gathered from both intervention and control group participants. Three months is considered as a sufficient time for the first follow up due to the intensity of the service and the functional capabilities of the participants. The follow-up questionnaire is a short version of the baseline survey including relevant measurements and questions for examining the effectiveness of day activity service in terms of the outcome measures. If the study participants do not return the follow-up survey, they will be called and encouraged to continue the study if possible. Throughout the study, the study participants are offered assistance in filling in the survey in case of visual impairment, for example. At the time of follow-up surveys, IG participants will also be interviewed with ASCOT-tool (Fig. 1.)

#### Outcomes and variables

The primary outcome of the trial is social inclusion, which is measured with the Experiences of Social Inclusion Scale (ESIS) [20]. Secondary outcomes are loneliness and social care related quality of life. Loneliness is measured with De Jong Gierveld Loneliness Scale [21] and single question: How often do you feel lonely? The response options for the single question are: “never”, “very rarely”, “sometimes”, “fairly often”, and “all the time”. Social care related quality of life is measured with Adult Social Care Outcomes Toolkit (ASCOT) [22, 23]. In addition, exploratory outcomes are social networks (LSNS-6 [24]), social support (MOS-SSS [25]), sense of security (single question: How safe are you feeling your life now?), unmet needs related to activities of daily living and instrumental activities of daily living [3] and general quality of life (EUROHIS-QOL 8-item [26]).

Perceived effectiveness is measured in the follow-up surveys for IG participants with two questions: 1. How useful do you find the day activity service for yourself? 2. How meaningful do you find the day activity service for yourself? A five-point Likert scale is used with the following response options: “Very useful/meaningful”, “useful/meaningful”, “neither useful/meaningful or useless/meaningless”, “useless/meaningless”, “very useless/meaningless”.

Changes in life situation or participation during the trial are asked about in the follow-up surveys. Indicators are health or functional ability status, family situation, social relationships, hobbies or other activities, and other changes. Options for answers are “No changes”, “Yes, positive changes”, “Yes, negative changes” and “Yes, both positive and negative changes” (see [27]).

Background variables are collected only in the baseline survey including age, sex, education, income, marital status, native language, living environment, form of housing, cohabitation status, and informal caring status. Other variables and measurements used in the baseline survey are material deprivation (seven items [28]), ability to make ends meet, access to services (health and social care, transport, other services), adequacy of social and health care services, access and use of internet and digital devices, environmental and neighbourhood satisfaction, safety and security in home and surrounding environment, ageism, abuse [29], and access and participation in voting.

Information about the intervention group members’ participation in the day activity service is provided by the municipalities after the six-month trial period.

#### Statistical analysis

First, descriptive analyses will be conducted to indicate similarities and differences between baseline characteristics in the IG and in the CG. The chi-square test is used to compare categorical variables. Continuous variables will be compared using t-test (normal distribution) and Mann-Whitney U test (non-normal distribution).

Intention-to-treat analyses will be performed primarily and for all outcomes. In addition, per-protocol analyses are used in a supplementary sense. Results of the categorical outcomes will be presented through percentages and continuous outcomes through mean scores and standard deviations or medians and quartiles. To evaluate the effectiveness of the intervention, the data will be analysed using suitable methods for correlated panel data such as mixed effect modelling or generalized estimating equations modelling [30]. Methods of multiple imputation will be used if necessary [31]. The challenges of non-randomization will be taken into account using statistical methods, such as propensity score weighting [32], if necessary.

Subgroup analyses will be performed for age, sex, frequency of participation, research area (two areas) and duration of the intervention (2–4 hours/6 hours). In addition, knowledge on tailoring the group activities learned from interviews with professionals (see Process evaluation) will be utilized in the analyses if necessary.

The data will be reported following the CONSORT guidelines [33].

### Economic evaluation

#### Study conduct

The economic evaluation (cost-effectiveness analyses, CEA) is part of the quasi-experimental trial design. Therefore, the study population and selection criteria will be the same as described previously.

#### Costs

The administrative data of social and health care service use and the costs of the intervention will be utilized in examining the cost-effectiveness of the intervention [34]. Costs of the day activity service will be retrieved from the service providers. Costs of health and social services will be calculated based on service use and the unit costs of the services [35].

#### Outcomes

Outcome measures for social inclusion, loneliness and social care related quality of life are the same as described in previous section.

#### Statistical analysis

The cost-effectiveness of the intervention will be analysed in relation to home care without day activity service using the incremental cost-effectiveness ratio (ICER) [36]. The ICER indicates between-group differences in costs and outcomes in 3- and 6-months follow-up periods, and will be calculated as follows:

$$\textrm{ICER}=\left({\Delta \textrm{C}}_{\textrm{IG}}-{\Delta \textrm{C}}_{\textrm{CG}}\right)/\left({\Delta \textrm{E}}_{\textrm{IG}}-{\Delta \textrm{E}}_{\textrm{CG}}\right)$$

where

∆C_IG_ = change in mean costs in the intervention group.

∆C_CG_ = change in mean costs in the control group.

∆E_IG_ = change in mean effectiveness in the intervention group.

∆E_CG_ = change in mean effectiveness in the control group.

Bootstrap simulation will be conducted to describe the statistical uncertainty related with the observed ICER. The probability distribution of ICER will be examined graphically by cost-effectiveness planes and cost-effectiveness acceptability curves.

### Process evaluation

#### Participants, selection criteria and recruitment

In addition to the survey data collected during the trial, qualitative data will be collected to evaluate the intervention process. The qualitative methods will include individual interviews with a sub-group of intervention participants (10 interviews) and focus group interviews with professionals (mostly practical nurses) tutoring the day activity service groups and involved in delivering older people’s social and health care services (4 focus groups of 4–6 persons). Participants for the individual interviews will be recruited from the IG. Recruitment will take a pragmatic approach to identify participants from both urban and rural areas, as well as participants with varying physical functional abilities and the number of participations in the day activity service. Tutors of the day activity service will be recruited from each municipality or location where the intervention is carried out. Other professionals (home care workers, dementia care nurses, for example) will be invited to focus group interviews based on discussions with home care professionals and leaders about their intervention delivery experiences, to ensure that a broad range of experiences are represented.

#### Study conduct

Individual interviews with the intervention participants will be conducted at the end of the six-month trial. The interview will be composed of three parts: 1) Understanding the participant’s functional ability, needs and services utilised (perceptions of their life situation and coping in every-day life, formal services and support in use, informal support, details and reasons of participating the day activity service), 2) General experiences of the day activity service (description of the day activity service, expectations, the most important activities/contents, perceived benefits and disadvantages, essential factors of successful service, barriers to participation, experiences of social inclusion and client-centredness, development ideas), 3) Experiences of the components of the day activity service (social activities, physical activities, other activities, tutors, lunch, information and advice) to explore how the components contribute to the overall intervention.

Focus group interviews with professionals will be conducted after halfway through the trial. Focus group interviews include three main sections: 1) Experiences of delivering the day activity service (aims, contents, duration, and participants of the intervention in different areas, any adaptations or tailoring made during delivery), 2) Factors affecting the implementation (organizational, professional and client-related barriers and facilitators of implementation), 3) Development of the intervention and understandings of how the intervention and its components are intended to work (program theory). The first section will include consideration of how different practices adopt the intervention and any local adaptations made. This knowledge related to tailoring/adaptation will be utilized in statistical analyses of the quasi-experimental trial. For example, by grouping participants based on major differences in tailoring of the day activity group they attend.

#### Analysis

Collection and analysis of qualitative data will be carried out iteratively where possible to ensure that important aspects appearing in early interviews can be considered in later interviews (see [16]). Thematic analysis will be used to analyse the interview data. First, two researchers will carry out the preliminary data coding separately, and then the final categories will be agreed together.

### Ethics

The research is carried out following guidelines of the Finnish Advisory Board on Research Integrity (TENK). Thorough ethical consideration is an important part of the process throughout its different phases: from recruitment to data collection, storage of data, analysis, and reporting. Participation in the research is voluntary and participants are given detailed information about the study at the recruitment phase. An informed consent form is obtained and confirmed from each participant individually regarding all parts of the study. Throughout the study, participants are encouraged to contact the researchers if any questions or doubts come up. The data collection is carried out with respect and discretion, and the reports will not reveal the identity of the participants. The research has received a supportive statement based on an ethical pre-assessment by the research ethical committee of the University of Eastern Finland. Research permissions have also been applied and granted by the study sites (one municipality and one social welfare and health care joint authority consisting of seven municipalities). The study sites are covered under the ethical approval provided by the committee of the University of Eastern Finland.

## Discussion

### Strengths and limitations

To our knowledge, this is the first study examining the effectiveness and cost-effectiveness of the day activity service targeted at older home care clients in Finland. Hence, the study offers novel understanding on the possible benefits of this type of intervention in addressing old-age social exclusion. In addition, this is a mixed method trial not only focusing on effectiveness but also evaluating the process of the intervention. Using a mixed methods approach, it is possible to measure effects of the intervention quantitatively, as well as explore experiences of the study participants qualitatively. We argue that it is important to highlight the voices and perceptions of older home care clients in relation to the day activity service.

A limitation of the study is the non-randomization especially in case of challenges on forming the comparison group. The challenge may be to find enough (*n* = 100) home care clients who are lacking social activities such as peer group activities. In addition, non-randomization may cause differences in the main sociodemographic and other background variables between groups at baseline. However, possible differences will be considered in statistical analyses.

### Implications

The study aims to produce comprehensive understanding of the effectiveness of the day activity service. The knowledge of the effects on older people’s social inclusion, loneliness and social care related quality of life will indicate whether there is need to redesign the existing intervention or develop novel interventions to reduce or alleviate social exclusion of home care clients. The results of the process evaluation can be utilized to form an advanced program theory of the day activity service to inform future implementation or refinements.

The results of the economic evaluation will be useful for decision makers directing scarce resources to services which provide effectiveness by reasonable amount of resources.

## Data Availability

The datasets generated during and/or analysed during the present study will be available from the corresponding author on reasonable request. The administrative data that support the findings of this study are available from Findata but restrictions apply to the availability of these data, which will be used under license for the current study, and so are not publicly available.

## Abbreviations

ASCOT: Adult Social Care Outcomes Toolkit
CG: Comparison Group
ESIS: Experiences of Social Inclusion Scale
ICER: Incremental cost-effectiveness ratio
IG: Intervention Group
