# Correction to: Study protocol of a mixed method pragmatic quasi-experimental trial to evaluate the day activity services targeted at older home care clients in Finland

**DOI:** 10.1186/s12877-023-03872-8

**Published:** 2023-04-20

**Authors:** Hanna Ristolainen, Leena Forma, Jemma Hawkins, Elisa Tiilikainen

**Affiliations:** 1 University of Eastern Finland, Yliopistonranta 1, 70211 Kuopio, Finland; 2 Laurea University of Applied Sciences, Ratatie 22, 01300 Vantaa, Finland; 3 Faculty of Social Sciences and Gerontology Research Center (GEREC), Tampere University, Kalevantie 4, 33100 Tampere, Finland; 4 Cardiff University, Spark, Maindy Road, Cardiff, CF24 4HQ UK

**BMC Geriatrics (2022) 22:810**

10.1186/s12877-022-03512-7

After publication of this article [1], the authors reported that the 3rd author (Hawkins) was incorrectly affiliated to affiliation 3, but this should have been affiliation 4, and the 4th author (Tiilikainen) was incorrectly affiliated to affiliation 4, but this should have been affiliation 1.

The original article [1] has been corrected.
